# Study of the Effect of Cedar Sawdust Content on Physical and Mechanical Properties of Cement Boards

**DOI:** 10.3390/molecules29184399

**Published:** 2024-09-16

**Authors:** Anas El Hamri, Yassine Mouhib, Atmane Ourmiche, Mohammed Chigr, Nour-Eddine El Mansouri

**Affiliations:** Laboratory of Molecular Chemistry, Materials and Catalysis (LC2MC), Faculty of Science and Technology of Béni-Mellal, University Sultan Moulay Slimane, B.P. 523, Béni-Mellal 23000, Morocco; yassinemouhib.dr@gmail.com (Y.M.); aourmiche@gmail.com (A.O.); chigrm@gmail.com (M.C.)

**Keywords:** cedar sawdust, wood cement boards, bio-based material, mechanical properties, physical properties, cement hydration products

## Abstract

The growing demand for sustainable building materials, amid escalating costs, has spurred interest in alternative solutions such as wood cement composites. This study explores the feasibility of producing wood cement boards (WCBs) using locally sourced cedar sawdust as a reinforcing agent. Boards with a thickness of 10 mm and a target density of 1200 kg/m^3^ were manufactured under pressures ranging from 2 to 6 MPa for 24 h. Cedar sawdust, used as raw and untreated material, was incorporated into the mixture as a partial substitute for cement in varying proportions, ranging from 10% to 25% (by weight). The WCBs were cured for 28 days under ambient conditions. Physical properties including density, water absorption (WA), and thickness swelling (TS) were assessed, along with mechanical properties through flexural tests. The results showed that increasing cedar sawdust content decreased both density and mechanical performance while increasing WA and TS. Microstructural analysis (SEM and EDS) revealed significant porosity at higher sawdust contents, while lower contents had better matrix–reinforcement cohesion. Additionally, substantial levels of calcium and silicon were detected on the sawdust surface, indicating stabilized cement hydration products. These findings, supported by thermal (TGA and DSC) and FTIR analyses, clearly demonstrate that cement boards with 10% cedar sawdust exhibit favorable properties for non-structural applications, such as wall and partition cladding.

## 1. Introduction

Recent decades have witnessed a global scientific renaissance aimed at addressing the increasing demands of everyday life. However, this advancement has often compromised natural heritage and ecological stability, especially in developing countries. In these regions, factors such as population growth, overgrazing, logging, and fuelwood collection have led to forest degradation and, ultimately, deforestation. For instance, logging activities have diminished Morocco’s cedar forests by 12%, threatening its ecological balance [1]. The cedar, a genus of conifers in the Pinaceae family, is native to the mountains of the western Himalayas and the Mediterranean region [2]. It encompasses several species, including the Atlas cedar (*Cedrus atlantica*), which is endemic to northwest Africa, specifically Morocco and Algeria [3]. The Atlas cedar plays a vital role in Morocco’s forestry sector, contributing to 80% of the nation’s timber production [4]. Despite its ecological and economic significance, the Atlas cedar population continues to decline due to unsustainable practices. This decline persists even as international organizations like UNESCO recognize Morocco’s cedar forests as vital biosphere reserves essential for global biodiversity. These challenges underscore the urgent need for innovative strategies to alleviate pressure on forest resources and enhance sustainable management practices [5]. Such an approach can help preserve forest biodiversity, stabilize ecosystems, mitigate environmental erosion, and combat climate change [6].

The growing interest in sustainable building materials has driven research into the utilization of lignocellulosic waste from agroforestry industries [7]. In Morocco, sawmills generate over 100,000 tons of sawdust annually, yet its utilization remains minimal [8]. Typically, sawdust is used in low-value applications, such as fuel, industrial site cleaning, or bedding for poultry and livestock, which do not fully exploit its potential as a valuable resource. To better harness this potential, recent research has shifted focus toward transforming sawdust into a functional material for construction, particularly through the production of wood cement boards (WCBs) [9].

WCBs are composite materials made by combining wood particles (aggregate), Portland cement (mineral binder), chemical additives, and water under pressure [10,11]. These boards offer an environmentally friendly alternative to traditional wood or asbestos cement boards [12]. Among the various types of mineral-bonded wood composites, cement-bonded particleboard is prominent, consisting of 10–70% wood particles and 30–90% Portland cement binder [13]. Woody material residues are often used as aggregates and include planer shavings; sawmill residues such as slabs, edges, and upholstery; logging residues from furniture match factories; and deforestation residues such as short trunks, broken logs, bent trunks, small crowns and branches, forest thinning, and bark [14]. Over the past few decades, numerous types of composite materials have emerged, including cement-bonded wood wool (CBWW), cement-bonded particleboard (CBPB), and fiber-reinforced cement board [15]. While resin-bonded boards are an alternative in some applications, the high cost of resins and the machinery required for production point in favor of the cement-bonded board [7]. Moreover, replacing organic adhesives like phenols and formaldehyde with cement-based adhesives aligns to manufacturing safe, environmentally-friendly products.

The incorporation of wood particles into the cement matrix inhibits crack propagation, enhancing fracture toughness, ductility, and flexural strength, which allows the board to withstand higher deformation limits, making it more resilient and durable in various applications [16,17]. The quality of these composites is influenced by various factors such as the type of lignocellulosic material used, density, particle size and volume, aspect ratio, mix design, mixing method, and treatment processes [18]. WCBs have a wide range of applications, including exterior wall cladding surfaces, siding panels, roof shakes, fire protection elements, specialized flooring, thermal and acoustic insulation purposes, building renovations, decorative applications, and sound insulation [10]. Their adaptability allows them to be molded into any shape to meet specific needs [6]. Furthermore, WCBs are asbestos-free, are devoid of hazardous and volatile substances, and minimize the spread of dust and toxic gases, ensuring a safe and environmentally friendly manufacturing process [19]. Cement-bonded boards are also highly resistant to various factors including freeze, thaw, fire, water, rot, termites, insects, and fungi attacks [6]. Their accessibility, low density, light weight, rigidity, non-corrosive nature, workability, low cost, and ease of manufacturing make them highly versatile and promising for a wide range of applications [20].

While there is a growing body of literature on the use of sawdust in composite materials, several gaps remain in the research. The careful selection of wood type is crucial for WCB production to ensure good chemical compatibility with cement and minimize chemicals hindering adhesion. Coniferous wood species, such as cedar, are commonly favored due to their superior chemical compatibility with cement [9]. Some wood species may impede cement hydration due to the type and content of extractives they contain [21]. Wood particles can be treated with hot water, sodium hydroxide, or other chemicals to address this. Additionally, mineralizing agents like calcium chloride (CaCl_2_) can be added to the mix to accelerate cement setting [22]. However, these procedures often increase the cost of production and energy consumption and result in a slower production process [23].

This study introduces a novel application for Atlas cedar residues by utilizing sawdust as a reinforcing material in WCBs. This approach not only enhances the aesthetic value and fragrance of the boards but also addresses the lack of research on the specific use of cedar sawdust. The primary objective of this research is to examine the impact of varying cedar sawdust content on the physical, mechanical, morphological, and thermal properties of WCBs without any additional chemical modifications. This study contributes to the development of sustainable building materials that minimize environmental impact, improve resource efficiency, and promote forest conservation. The insights gained from this research can serve as the basis for optimizing WCBs in diverse non-structural applications, such as wall and partition cladding.

## 2. Results and Discussion

### 2.1. Chemical Constituents of Cedar Sawdust

Several challenges still significantly hinder the advancement of wood–cement composites, notably the substantial variability in the compatibility of different wood species with cement constituents and the inhibitory impact that certain wood species have on cement’s hydration [24]. Certain wood components, such as holocellulose, lignin, and total extractives, can impede the bond between wood particles and cement, potentially delaying cement hydration or setting time. Conversely, substances like ash can enhance compatibility between these elements [25].

To ensure successful wood–cement composite production, it is crucial to evaluate the chemical composition of wood species and select those with minimal elements hindering the bond between wood and cement molecules. In this study, the suitability of cedar sawdust for cement board manufacturing was evaluated by comparing its chemical composition, based on the literature (Table 1) [26], with that of certain previously used species in these boards. The conifer family, to which cedar belongs, is often preferred for the production of wood–cement boards (WCBs) due to their generally more chemically compatible nature with cement, as they do not contain substances that inhibit its hardening [9].

Wood compositions primarily contain organic chemical compounds such as cellulose, hemicelluloses, and lignin, which collectively constitute over 99% of the total mass [27]. In this context, Pettersen et al. [28], reported that wood generally has a higher proportion of holocellulose than of lignin, extractives, and ash. Similarly, the chemical composition of Atlas cedar wood, as shown in Table 1, indicates a higher quantity of holocellulose (62.10%) compared to lignin (32%), extractives (4.24%), and ash (0.31%). These lignin, holocellulose, and ash contents are comparable to those of other species previously used in wood–cement composite manufacture, such as eucalyptus [11], pine [18], tropical woods [25], and Ochroma pyramidal [9]. However, the extractive content in Atlas cedar wood is lower than in most of these species.

The amount and composition of extractives are specific to each wood species and are the main reason for the prevention of cement hydration, leading to incompatibility between wood and cement [15,29]. Extractives are generally composed of terpenes, fatty acids, tannins, carbohydrates, and inorganic substances [30]. They also contain several organic compounds that form complexes with metal ions present in the cement solution, reducing the concentration of calcium ions (Ca^2+^), and disrupting the equilibrium of the solution, thereby delaying the onset nucleation of Ca(OH)_2_ and calcium silicate hydrate (C-S-H) gel [25].

As the content of these elements increases, so does the percentage of anhydrate clinker, resulting in reduced mechanical performance of wood–cement composites [15]. Hence, wood species with low levels of extractives are deemed more suitable for wood–cement composites [29]. For instance, Mandes et al. [18] found that boards produced from eucalyptus wood residues displayed better physical and mechanical properties because of their low extractives content (2.3%) in contrast to other wood sources such as Pinus wood, sugarcane bagasse, and bamboo particles, which had higher extractives contents of 10.8, 12.5, and 9.2%, respectively. Thus, the lower percentage of extractives (4.24%) in Atlas cedar wood is in line with the expected results of this study.

In previous studies, attempts were made to improve board properties by removing certain extractives to expedite the cement setting and hardening process. However, this treatment did not always yield beneficial results. For instance, Setter et al. [9] utilized Ochroma pyramidal wood with a comparable extractives content (5.78%) and applied treatments involving cold water, hot water, and NaOH solution. However, the outcomes did not demonstrate any enhancement in the final quality of the produced boards, whether in terms of physical or mechanical properties. Consequently, these findings have favored the use of cedar sawdust in its untreated form, reducing energy consumption and expediting the production process.

XRF analysis of the cement unveiled its chemical composition, comprising various metal oxides, as detailed in Table 2. Chowdhury et al. [31] highlighted that these oxides are also present in wood ash in significant quantities. This suggests that the cementitious properties of wood ash enhance the role of cement in the production of wood–cement composites, as pozzolanic reactions occur between wood ash (pozzolan), water, and calcium hydroxide released by cement hydration, increasing the amount of the cement bonding phase (C-S-H) and improving cement bond strength [25]. However, considering the composition outlined in Table 1, Atlas cedar contains a limited amount of ash (0.31%), thereby reducing its potential to improve the properties of the produced boards.

### 2.2. Physical Properties

#### 2.2.1. Density

**Figure 1:**
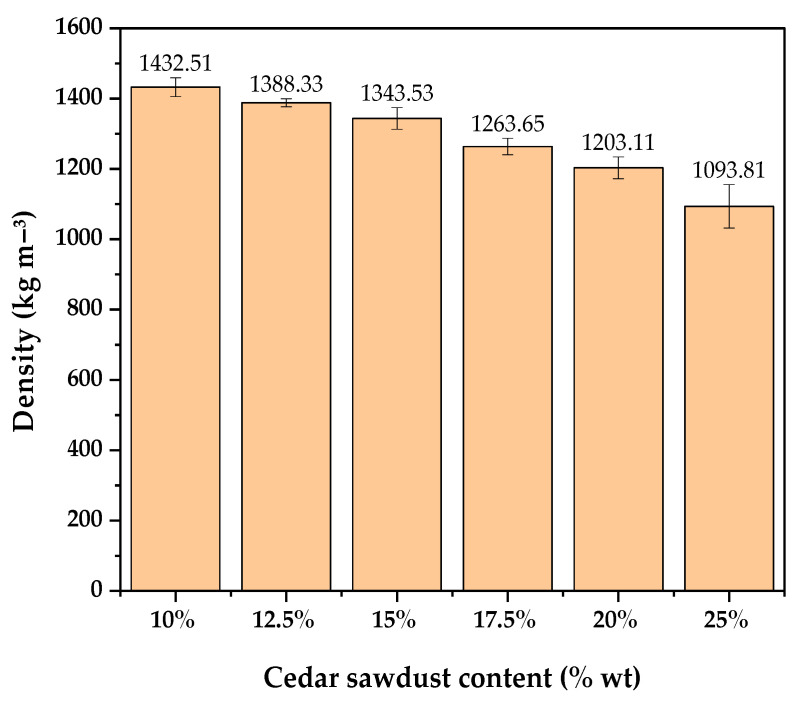
Variation in density with sawdust content.

The results of the physical and mechanical properties of the manufactured boards are shown in Table 3, while Table 4 reports their variance analysis. The change in density concerning cedar sawdust content, illustrated in Figure 1, indicates an average value ranging from 1093.81 to 1432.51 kg/m^3^. The lowest density was obtained for boards with a 10% sawdust content (WCB 1), while the highest density was observed in boards with a 25% sawdust content (WCB 6). The variance analysis for density revealed significant differences among all levels, indicating a noticeable effect of cedar sawdust content on board density.

As depicted in Figure 1, as the amount of cedar sawdust in the boards increased, there was a discernible drop in density. This trend aligns with results from some prior research, where an increase in the percentage of wood particles or fibers in wood–cement composites resulted in decreased density [32,33,34]. This decrease can be attributed to the higher proportion of cedar wood in the mixture, which has a much lower density (218 kg/m^3^) compared to cement (3047 kg/m^3^). In other words, increasing the amount of sawdust increases the total volume of the mixture required to produce a particleboard of a given thickness. It is essential to note that the mechanical and physical properties of particleboard can be influenced by board density and particle size [35].

Similarly, in the current study, it was observed that the board with the highest density value at 10% cedar sawdust content exhibited the highest modulus of rupture (6.94 MPa) and modulus of elasticity (3181.33 MPa), while the lower mechanical properties of the board prepared with 25% cedar sawdust may be attributed to its lower density. Except WCB 6, all of the produced boards meet the requirements for HZ-type BISON boards, which specify values not less than 1200 kg/m^3^ [36].

**Table 3:** Mean values obtained for physical and mechanical properties of cedar–cement boards.

Board	Density (kg/m^3^)	WA 2 h (%)	WA 24 h (%)	TS 2 h (%)	TS 24 h (%)	MOR (MPa)	MOE (MPa)
WCB 1	1432.51 (27) ^a^	11.11 (0.59) ^a^	12.35 (2.58) ^a^	0.27 (0.04) ^a^	0.36 (0.09) ^a^	6.94 (0.67) ^a^	3181.33 (286) ^a^
WCB 2	1388.33 (11) ^ab^	13.01 (1.79) ^ab^	13.70 (1.67) ^ab^	0.39 (0.04) ^b^	0.49 (0.11) ^a^	6.47 (0.75) ^ab^	2634.16 (188) ^b^
WCB 3	1343.53 (31)^b^	13.82 (1.24) ^b^	16.49 (2.31) ^b^	0.43 (0.04) ^bc^	0.65 (0.06) ^b^	5.21 (0.96) ^bc^	2197.83 (271) ^b^
WCB 4	1263.65 (23) ^c^	14.82 (0.55) ^b^	16.07 (1.32) ^b^	0.48 (0.02) ^c^	0.66 (0.06) ^b^	4.19 (0.82) ^cd^	1655.33 (350) ^c^
WCB 5	1203.11 (31) ^d^	17.77 (1.44) ^c^	21.07 (2.12) ^c^	0.67 (0.04) ^d^	1.16 (0.12) ^c^	3.65 (1.06) ^d^	1608.80 (414) ^c^
WCB 6	1093.81 (62) ^e^	23.06 (1.45) ^d^	27.75 (2.69) ^d^	0.71 (0.05) ^d^	1.22 (0.11) ^c^	1.27 (0.74) ^e^	476.83 (259) ^d^
BISON board type HZ [36]	1200	--	--	0.8	1.2–1.8	9	3000

Means followed by the same letter in the same column are statistically equal according to the Tukey test with a probability of 95%. Values in parenthesis are standard deviations.

**Table 4:** Variance analysis of physical and mechanical properties of cedar–cement boards.

Source	Density	WA 2 h	WA 24 h	TS 2 h	TS 24 h	MOR	MOE
Sawdust content	***	***	***	***	***	***	***

*** (significant on α < 0.01).

#### 2.2.2. Water Absorption and Thickness Swelling

During the lifespan of wood and its derivatives, exposure to weathering and various decomposition factors, both biotic and abiotic, significantly impacts their performance [37]. These factors, along with others, contribute to increased wood permeability and decreased resistance to water [38], underscoring the importance of assessing the durability of produced boards against such conditions. Water absorption (WA) and thickness swelling (TS) are crucial physical properties utilized to evaluate the dimensional stability of boards, particularly those intended for outdoor use under extreme humidity conditions [34,39].

**Figure 2:**
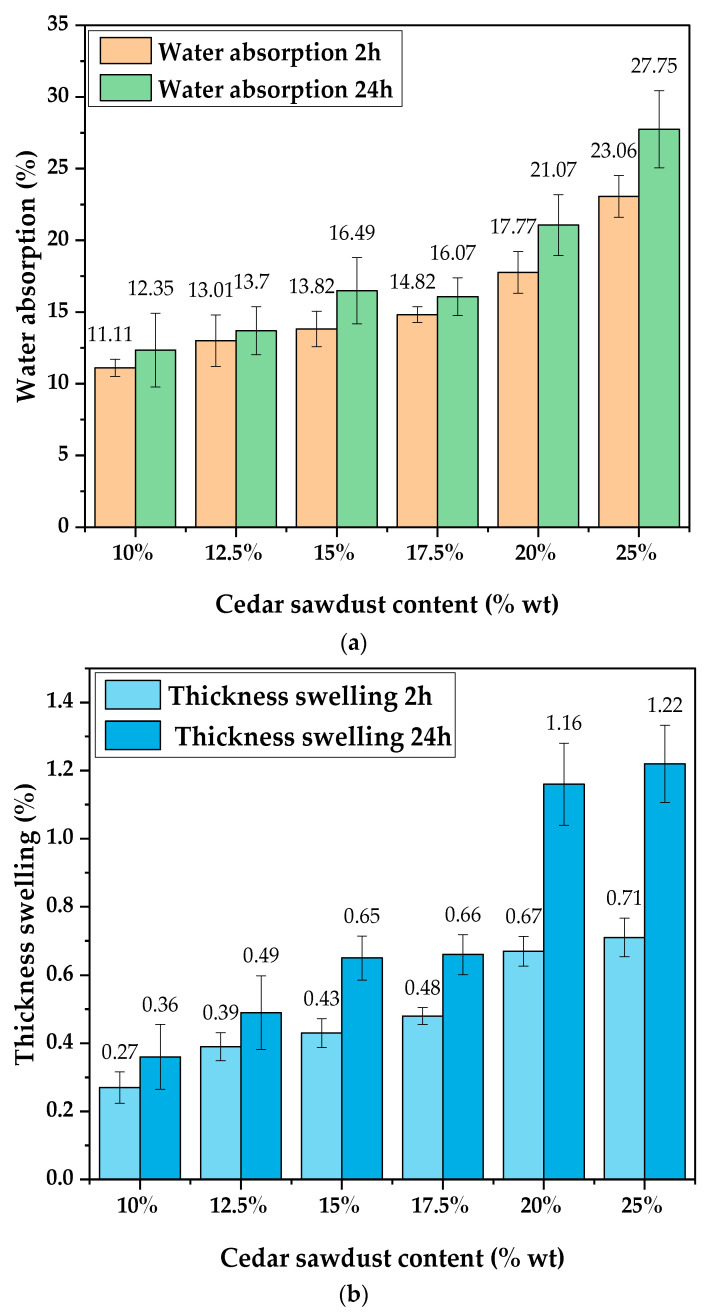
(**a**) Variation in WA with cedar sawdust content; (**b**) Variation in TS with cedar sawdust content.

This study investigates how sawdust content affects the WA and TS characteristics of the boards. Figure 2 provides the average %WA and %TS values after immersing the samples in water for both 2 h and 24 h. Notably, boards with 10% sawdust (WCB 1) display the lowest WA and TS values across both immersion durations, while those containing 25% sawdust (WCB 6) exhibit the highest values. Specifically, Figure 2a shows WA values ranging from 11.11% to 23.06% after 2 h of immersion and from 12.35% to 27.75% after 24 h. However, Figure 2b reveals that average TS values vary from 0.27% to 0.71% after 2 h of immersion, and from 0.36% to 1.22% after 24 h. The increase in both WA and TS with immersion time is evident, as prolonged immersion increases the likelihood of water permeating the board’s interiors, heightening their instability. This phenomenon can be attributed to the inherent hygroscopic properties of wood fibers, which possess a considerable affinity for water [40]. This aligns with findings from Setter et al. [9], where increasing the soaking time of WCBs from 2 h to 72 h resulted in similar trends.

Additionally, statistical analysis of the results in Table 3 reveals that these properties intensify with higher sawdust content in the boards, particularly noticeable for WCB 5 and WCB 6. Other researchers have reported similar trends. For instance, Ali et al. [41] observed an increase in both WA and TS as the sawdust content in cement boards ranged from 20% to 40%.

Likewise, Bilcati et al. [42] conducted an evaluation of cement composites with sawdust content between 10% and 20%, supporting these findings. In addition to the hygroscopic nature of lignocellulosic materials and the composition of its hemicellulose-rich fibers, which are inherently more hydrophilic [43], this type of behavior can also be explained by the wood particles’ porosity [41]. Sotannede et al. [34] pointed out that the difficulty in compressing and the presence of void spaces in the boards enhance water absorption. Hence the high proportion of wood particles within the board increases the rate of pores accessible to water, accounting for the low densities recorded at high sawdust content.

Conversely, increasing the percentage of cement and decreasing the sawdust content reduces the water absorption by the boards [34]. This effect occurs because the binder fills the pore holes that would otherwise result from the wood’s porosity [19]. For sawdust content ranging from 10% to 20%, the WA values observed in this study are lower than those reported by other researchers. For example, Olorunnisola et al. [44] used eucalyptus wood particles as reinforcement, while Bilcati et al. [42] assessed lightweight cement-based composites reinforced with Pinus sawdust. Conversely, for a 25% sawdust content (cement–wood ratio of 3:1), several studies found WA values lower than those observed in this study (27.75%). For instance, Ogunjobi et al. [19], using Anogeissus leiocarpus wood particles, reported an average value of 24.95%. Similarly, Adelusi et al. [45] and Amiandamhen and Izekor [46] found values of 24.45% and 21.59%, respectively, using Gmelina arborea sawdust in cement-bonded boards.

The variation in results observed for the 25% sawdust content compared to previous studies could be attributed to particle size effects. Smaller dimensions lead to increased surface area of the particles, making it challenging for the cement to adequately cover them all. To ensure the production of high-quality cement-bonded boards with good dimensional stability, it is recommended to use mixed-size or coarse particles [13]. Mendes et al. further substantiated this point by confirming that particle size and geometry significantly impact the physical properties of the cement-bonded boards, with larger particles yielding better performance [18]. Similarly, Sotannde et al. demonstrated that composites made from a mixture of sawdust and flakes outperformed those made from each component separately [34]. In this study, particle size diversity was limited (ranging from 2 mm to 4 mm). Additionally, there may have been an incompatibility between wood and cement, resulting in internal free space at the particle–cement interface, which potentially led to significant moisture absorption. Frybort et al. highlighted that the presence of extractives is one of the primary causes of this incompatibility [15].

In prior research endeavors, chemical and/or physical pretreatment involving additives has often been employed during the manufacturing process of cement-bonded boards to enhance their dimensional stability. For example, Sotannde et al. explored the impact of incorporating three different chemical additives (CaCl_2_, MgCl_2_, AlCl_3_) on the WA and TS of CBPB derived from Afzelia Africana wood residues. Their findings revealed that the introduction of chemical additives led to a reduction in water absorption of the boards by up to 16% [34]. Similar conclusions have been reached by other studies as well [41,47]. The average TS values obtained in this study closely align with the findings of most of the previously mentioned works [41,44,45,46]. They also conform to the normative standards outlined by both the CBPB manufacturer Cetris^®^ [48], and the requirement set by BISON for an HZ-type board [36] which stipulates a maximum of 1.8% TS. However, only the WA values of WCB 1, WCB 2, and WCB 3 are close to those specified by the manufacturer Cetris^®^ (16%).

### 2.3. Mechanical Properties

**Figure 3:**
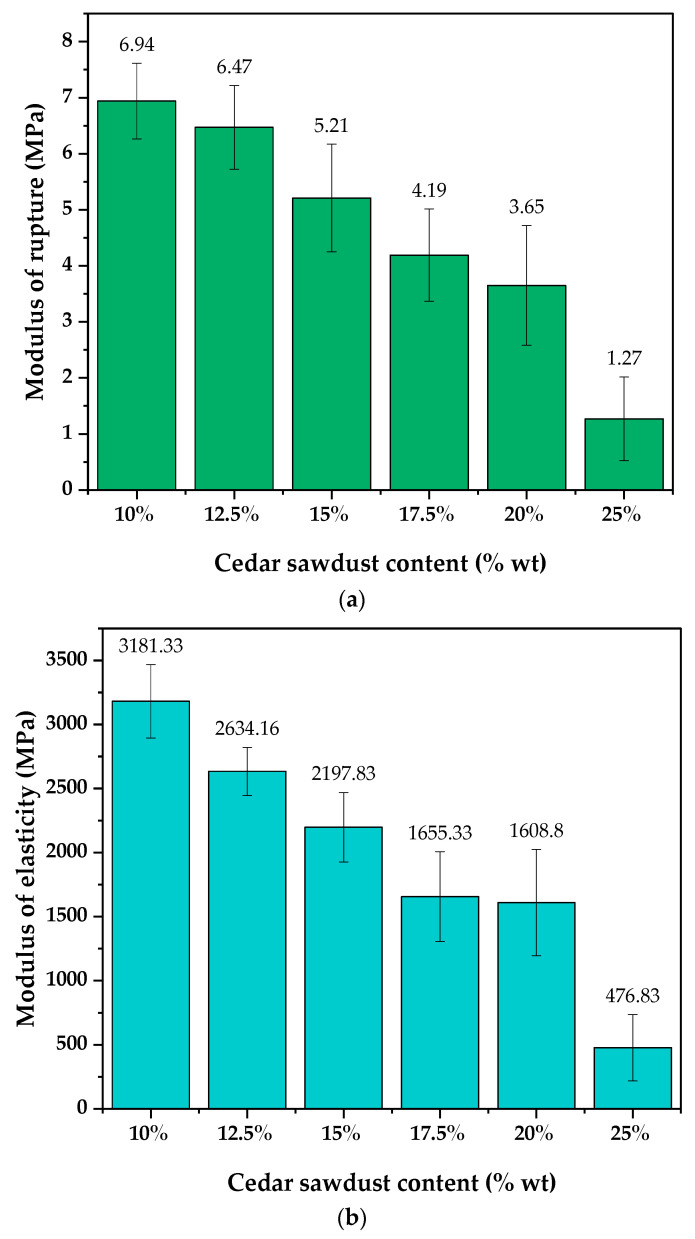
(**a**) Variation in MOR with cedar sawdust content; (**b**) Variation in MOE with cedar sawdust content.

The flexural mechanical properties of the produced boards were evaluated through measurements of the modulus of rupture (MOR) and the modulus of elasticity (MOE). As illustrated in Figure 3, MOR values ranged from 1.27 to 6.94 MPa, while the MOE values varied from 476.83 to 3181.33 MPa. A thorough statistical analysis of the results in Table 3 indicates that cedar sawdust content significantly affects mechanical properties. Notably, there is a discernible negative correlation between sawdust content and board strength: as cedar sawdust content increases, mechanical properties decrease. Boards containing 10% cedar sawdust (WCB1) exhibited the highest values of MOR (6.94 MPa) and MOE (3181.33 MPa), aligning with previous research on cementitious composites using various lignocellulosic reinforcements, such as eucalyptus [44], oil palm veins [49], and rattan particles [50], where optimal mechanical properties were similarly achieved at 10% content. However, other studies have reported maximum mechanical properties with reinforcement contents exceeding 10% [51,52]. These results can be attributed to several factors, including improved compatibility between particles and the cementitious matrix, effective particle dispersion within the cement matrix, and the pressing cycle during board production, which minimized voids as much as possible [40].

Beyond 10% cedar sawdust content, mechanical properties declined, with a more pronounced decrease observed when content exceeded 20%. This trend mirrors findings from some previous studies. For instance, Ali et al. noted a 17% to 48% decrease in strength properties when sawdust content increased from 20% to 30% and 40% in cement particleboards [41]. Similarly, Ayrilmis et al. noted a decline in the mechanical properties of cement-bonded composites with increased palm particle main vein content beyond 10%, attributing this to increased composite porosity and reduced particle–matrix interfacial area [49]. The significant decrease in mechanical properties observed beyond 20% cedar sawdust content for WCB 6 can be attributed to inadequate compaction and the lower density of the wood–cement composites produced with high contents [33]. This phenomenon is linked to the attainment of high porosity due to a low cement-to-wood ratio, resulting in diminished MOE and MOR values [15]. At this juncture, the cement becomes insufficient to bind the wood particles, leading to maximum porosity, as evidenced by the low density (1093.81 kg/m^3^) recorded for boards with 25% cedar sawdust content.

Conversely, applying higher pressures during manufacturing leads to the densification of the wood, elimination of gaps, and stronger matrix–reinforcement connections, thereby enhancing strength and stiffness [53]. The findings of this study are comparable to those reported by Setter et al., who observed MOR values ranging from 3.08 to 3.58 MPa and MOE values spanning from 2197 to 2739 MPa when using Ochroma pyramidal wood particles as reinforcement in WCBs [9]. It is worth noting that the maximum mechanical property values of cement-bonded boards vary across different studies, primarily influenced by factors such as the aspect ratio, size, orientation, and interfacial adhesion of the particles used as reinforcement. Ghofrani et al. observed that for the same 25% reinforcement content, the MOR value obtained when incorporating hardwood fibers (8.1 MPa) was lower than that obtained with rice stalk fibers as reinforcement (8.6 MPa). They attributed this difference to the aspect ratio of the fibers, which is significantly higher in rice stalk fibers, enabling better stress transfer between the matrix and the fibers [52]. In another study, Nasser et al. achieved MOR and MOE values of 11.78 MPa and 5667 MPa, respectively, for cement boards produced using the prunings of certain Saudi Arabian tree species, highlighting the importance of employing mixed or coarse particle sizes to achieve high mechanical properties [13]. Similarly, Olorunnisola observed that boards prepared with 10% rattan particles of varying sizes exhibited a higher MOR value (6.8 MPa) compared to those with uniform particle size (4.3 MPa) [54].

Alternatively, in a study exploring the effect of strand orientation on the physical and mechanical properties of cement boards, Cabral et al. found that the best performance was observed with boards containing 35% parallel-oriented eucalyptus wood strands, with MOR and MOE values reaching approximately 15 MPa and 7072 MPa, respectively [55]. While cedar sawdust demonstrated effectiveness as reinforcement in WCBs, the mechanical properties achieved in this study were not on par with those reported in most previous research. This shortfall can be attributed to certain characteristics of the reinforcement employed, such as its uniform particle size, non-fibrous nature, low aspect ratio, and lack of orientation. These factors contribute to inadequate physical contact between particles.

To address these limitations and enhance the findings, it is essential to improve the properties of the reinforcement by adjusting particle size and orientation during the manufacturing process. Based on the findings of this study, none of the boards met the minimum bending strength (MOR) requirement of 9 MPa set by BISON for HZ-type boards. However, boards containing 10% cedar sawdust content surpassed the MOE requirements of 3000 MPa for HZ-type boards [36]. Despite not meeting structural standards, these boards remain suitable for applications where direct structural support is unnecessary, such as interior or exterior cladding for thermal and acoustic insulation purposes.

### 2.4. Microstructural Analysis

#### 2.4.1. Scanning Electron Microscopy (SEM)

**Figure 4:**
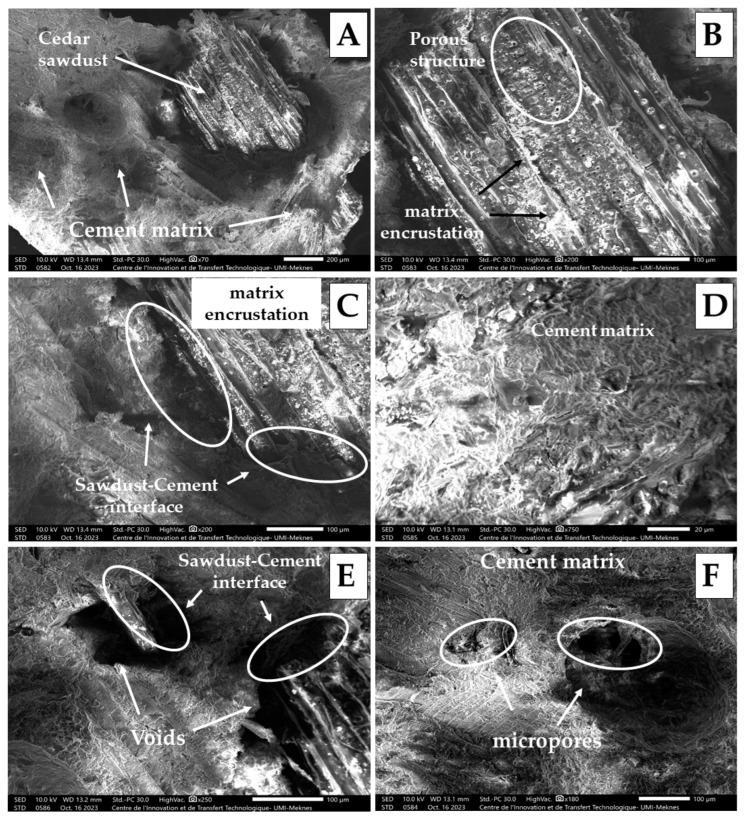
SEM micrographs of WCB samples: (**A**,**B**) the fractured surface; (**C**,**D**) board samples containing 10% cedar sawdust; (**E**,**F**) board samples containing 25% cedar sawdust.

Scanning electron microscopy (SEM) analysis stands as the predominant technique for scrutinizing fiber–matrix interactions at fracture surfaces and matrix distributions in plant fiber composites [56]. In this study, SEM was employed to unveil the internal microstructure and alterations on the fracture surface of the examined boards after the post-bending test, offering insights into some physical and mechanical properties obtained. Specifically, the analysis was conducted on two samples from each of the boards containing 10% and 25% cedar sawdust, with images captured at various magnifications, as represented in Figure 4.

The fracture surface unveils failure occurring either at the particle–matrix interface or within the cementitious matrix, with samples displaying fractured ends of sawdust particles and instances of pull-out from the matrix (Figure 4A), confirming that the interface is indeed the weakest part of the board, potentially due to the diminutive size of the particles used (2–4 mm). This mode of failure mirrors observations made by Amiandamhen et al. in cementitious composites containing wood residues, attributing it to the small size of the wood particles adversely affecting interfacial adhesion [56,57].

Samples containing 10% cedar sawdust content exhibit notable adhesion between the sawdust particles and the cement matrix, as evidenced by matrix encrustation on the surfaces of the sawdust particles (Figure 4B,C), indicating well-distributed particles devoid of agglomeration. The presence of matrix encrustation on fiber surfaces and the appearance of fractured ends of pulled-out fibers are clear signs of successful fiber reinforcement [58]. Furthermore, at this content, the matrix appears more compact and denser with reduced amounts of air pores at the interface (Figure 4D), fostering better adhesion between cedar sawdust and cement and resulting in superior physical and mechanical performance. Prior research attributes this cohesion to the formation of cement hydration products, which serve as anchors between reinforcement and the cement matrix, enhancing inter-particle conditioning in the composite [57,59,60]. Additionally, the quality of the cement significantly impacts the formation of these compounds, with studies demonstrating that the use of 52.5R Super White Cement generates more hydration products, thereby bolstering physical–mechanical properties due to its higher CaO and SiO_2_ content compared to 32.5R Portland cement [61].

Conversely, samples containing 25% cedar sawdust exhibit an escalation in porosity with the increase in cedar sawdust content (Figure 4E,F). The structure reveals the presence of air pores surrounding the wood particles at the interface (Figure 4E), while the cementitious matrix appears less dense and more hollow (Figure 4F). This suggests a weaker bond strength, with the cementitious matrix seemingly inadequate to fully bind all the wood particles together. This aligns with earlier research showing that higher aggregate content at low cement content results in inadequate hydration products, attributed to unreacted CaO and SiO_2_ [57]. Furthermore, Nasser et al. stated that voids within the particleboard may originate from air within the wood particles [13]. This assertion finds support in the microstructure of cedar sawdust illustrated in Figure 4B, validating their conclusions.

In summary, the porous microstructure of the used reinforcement affects board stability, with an increase in its content in the mixture explaining lower density, MOR, and MOE values, as well as higher water absorption observed.

#### 2.4.2. Energy-Dispersive X-ray Spectroscopy (EDS)

**Figure 5:**
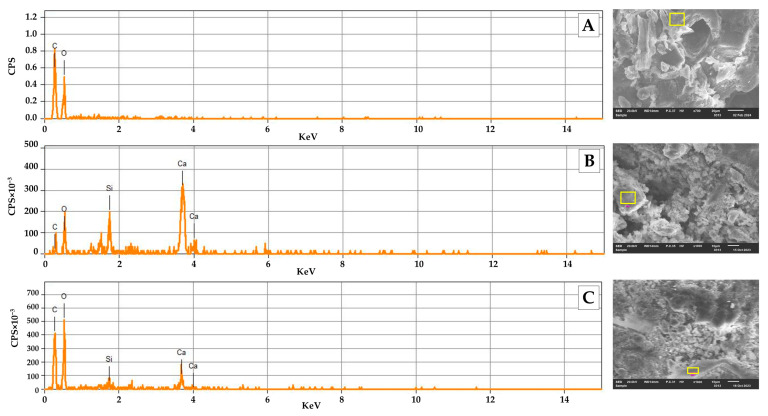
EDS spectra and analyzed points of (**A**) Cedar sawdust; (**B**) Cement matrix; and (**C**) Reinforcement.

Energy-dispersive X-ray spectroscopy (EDS) analysis was conducted on the cedar sawdust particles, as well as at two locations of the fracture surface of the board containing 10% cedar sawdust: on the surface of the cement matrix and the surface of the sawdust reinforcement. Figure 5A–C illustrate the EDS spectra obtained, respectively, while Table 5 provides a summary of the corresponding chemical weight composition.

Figure 5A reveals that cedar sawdust particles consist primarily of oxygen (O) and carbon (C), which are key components of lignocellulosic materials, while Figure 5B indicates that the cement matrix surrounding the reinforcement contains inorganic elements, such as calcium (Ca) and silicon (Si), as its main constituents. These findings align with the X-ray fluorescence (XRF) analysis presented earlier. The predominant element (Ca) originates from lime (CaO), while the silicon is derived from silica (SiO_2_). Some minerals, such as Al, Fe, and Mg, were not detected prominently due to the low proportions of alumina (Al_2_O_3_), hematite (Fe_2_O_3_), and magnesium oxide (MgO) in this type of cement.

Figure 5C reveals that the chemical composition of the reinforcement displays elevated levels of oxygen (O) and carbon (C) in comparison to the cement matrix, attributed mainly to the organic nature of the cedar sawdust particles utilized in board production. Additionally, the surface of the reinforcement exhibits a moderate presence of calcium and a relatively low quantity of silicon. This outcome is consistent with previous studies. For instance, Miranda et al. observed low amounts of Ca, Si, Al, and Mg on the surface of *Pinus* spp. Particles attribute this to the precipitation of materials from cement hydration on the cell walls of these particles [12].

Similarly, Brasileiro et al. noted a significant rise in Ca concentration on the surface of coir pith particles used as reinforcement in cementitious composites, attributing it to the ability of lignocellulosic materials to stabilize this element on their surfaces, along with the high mobility of Ca ions in the cement hydration solution. Consequently, there is an accumulation of cement hydration products, represented mainly by calcium hydroxide (Ca(OH)_2_) and hydrated calcium silicate (C-S-H), at the reinforcement–cement interface [59]. Aras et al. reported that the formation of these products increases as the amount of CaO and SiO_2_ components in the cement rises, strengthening the bonds between wood and matrix and improving the mechanical and physical properties of cement boards [61].

In addition to hydrogen bonds, cement hydration products contribute to the creation of a physical anchor between vegetal particles and the paste, due to the porosity of these materials. Furthermore, the analysis indicates a notable variation in the Ca/Si ratio at the fracture surface. Specifically, it increases from 3.52 at the fracture surface of the cement matrix to 4.05 at the surface of the reinforcement. This observation suggests that the localized augmentation in Ca/Si ratios at interfaces means that these regions facilitated the accumulation of more hydration products, such as CH crystals, thereby fostering the development of interlocking structures between wood and cement. Consequently, this phenomenon contributes to enhanced bond strength and elevated mechanical strength [60].

### 2.5. Fourier Transform Infrared Spectroscopy (FTIR)

**Figure 6:**
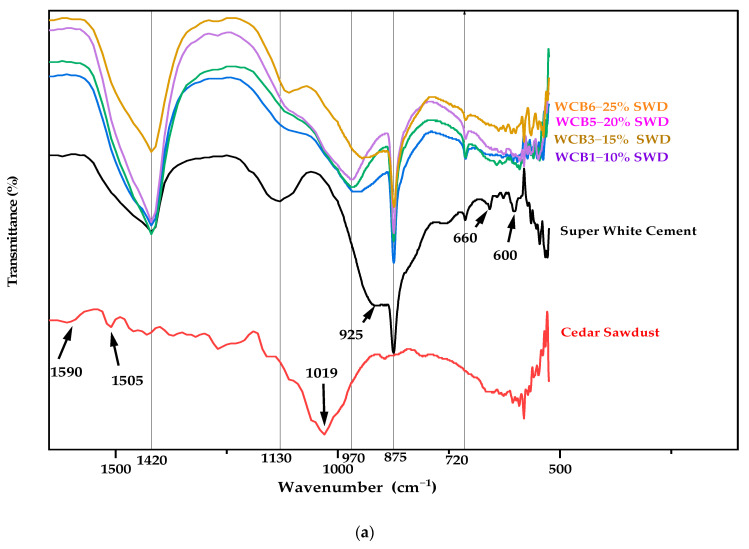

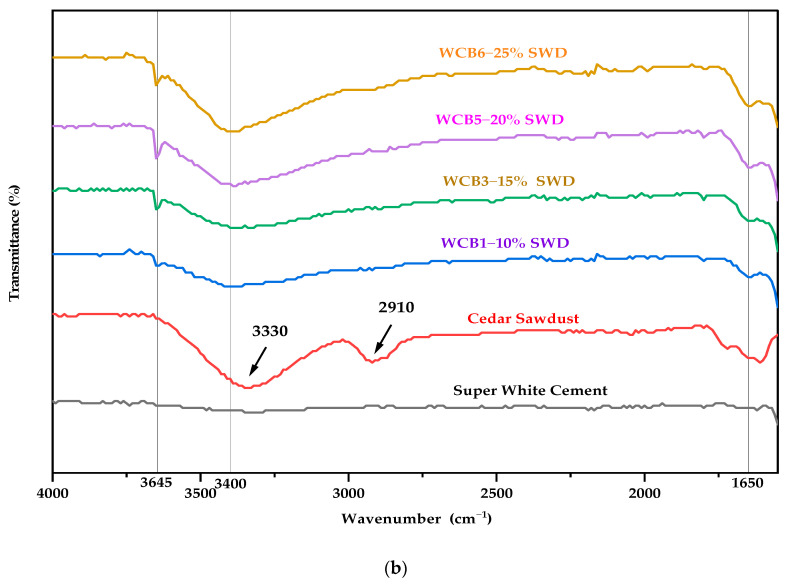
FTIR spectra of samples: (**a**) 1600–500 cm^−1^ (**b**) 4000–1600 cm^−1^.

Figure 6 displays the FTIR spectra for cedar sawdust, Super White Cement, and boards with different cedar sawdust contents (10%, 15%, 20%, and 25%). These results reveal several significant absorption bands that explain the findings of the MEB, EDS, and thermal analyses.

The FTIR spectra of the cement show intense peaks characteristic of the C-O bond in calcium carbonate (CO_3_^2−^), represented by an asymmetric stretching vibration mode at approximately 1420 cm^−1^ and a bending vibration mode at 875 cm^−1^ [62]. The band at around 925 cm^−1^ is related to the asymmetric stretching vibration of the silicate group (Si-O) in ${\mathrm{SiO}}_{4}^{4-}$, associated with the presence of the C_3_S and C_2_S mineral phases, and the band at 1130 cm^−1^ is attributed to the vibrations of the sulfate group (S-O) in ${\mathrm{SiO}}_{4}^{2-}$. The presence of these bands indicates the existence of calcium silicates and calcium sulfate such as gypsum and anhydrite [63]. The small peaks in the range of 712 to 780 cm^−1^ correspond to the symmetric stretching vibration of Si-O-Si, and those in the range of 600 to 660 cm^−1^ are attributed to the bending vibration of Si-O-Si [62].

The FTIR spectra of the cedar sawdust reveal the presence of a broad band located at 3330 cm^−1^, corresponding to the stretching vibrations of bonded O-H groups from the three main polymers present in wood (cellulose, hemicellulose, and lignin). The band at 2910 cm^−1^ is attributed to the asymmetric stretching vibration of aliphatic C-H bonds in methyl (CH_3_) and methylene (CH_2_) groups of holocellulose and lignin. The small bands at 1590 cm^−1^ and 1505 cm^−1^ correspond to the symmetric elongations of C=C bonds in aromatic rings, attributed to lignin, while the broad band located at 1019 cm^−1^ is attributed to the stretching vibration of the C-O bond of the primary alcohol in carbohydrates and the C-O-bond of ether in cellulose, or the CH_3_-O bond in lignin and the CH_3_-O- bond in ester [3,64].

The FTIR spectra of all the boards show a strong presence of the characteristic peaks of calcium carbonate at 1420 cm^−1^ and 875 cm^−1^. The intensity of these peaks increases with the cedar sawdust content in the boards, reaching its highest level at a cedar sawdust content of 20%. Govin observed similar results, noting that the intensity of the broad band associated with the C-O bond vibrations in calcium carbonate increased as the poplar content in the boards increased [65]. This increased formation of carbonates can be explained by the reaction of organic compounds present in the wood with the calcium in the cement, thus promoting the formation of calcium carbonates. This result corroborates the increase in carbonation observed during the thermal analysis.

Furthermore, the spectra reveal the increasing appearance of broad bands at 3400 cm^−1^ and 1650 cm^−1^, corresponding to the stretching and bending vibrations of bonded -OH groups and H_2_O, respectively [62]. The intensity of these bands rises with higher cedar sawdust content in the boards. This enhancement is likely due to the hydrophilic nature of organic compounds in the cedar sawdust, which promote the formation of calcium hydroxides (portlandite) during cement hydration, thereby increasing the presence of -OH groups.

The spectra reveal prominent bands at approximately 975 cm^−1^, associated with Si–O asymmetric stretching vibration. These bands confirm the formation of calcium silicate hydrate (C–S–H) gel, which is essential for the strength of the concrete [66]. Previous studies suggest that the peak associated with C-S-H gel in Portland cement systems represents a shift of the previously observed peak at 923 cm^−1^ in cement spectra to higher wave numbers [67,68,69]. Mollah links this shift to the polymerization of ${\mathrm{SiO}}_{4}^{4-}$ silicate units during the formation of C-S-H gel [69]. Additionally, isolated hydroxide ion vibrations were found to exhibit a distinctive fine band feature at 3645 cm. This band facilitates the monitoring of portlandite (Ca(OH)_2_) formation [63]. The intensity of this band slightly increases with sawdust content, suggesting that cedar sawdust promotes the precipitation of portlandite. This observation supports the noted increase in carbonation up to 20% cedar sawdust content.

### 2.6. Thermal Analysis

**Figure 7:**
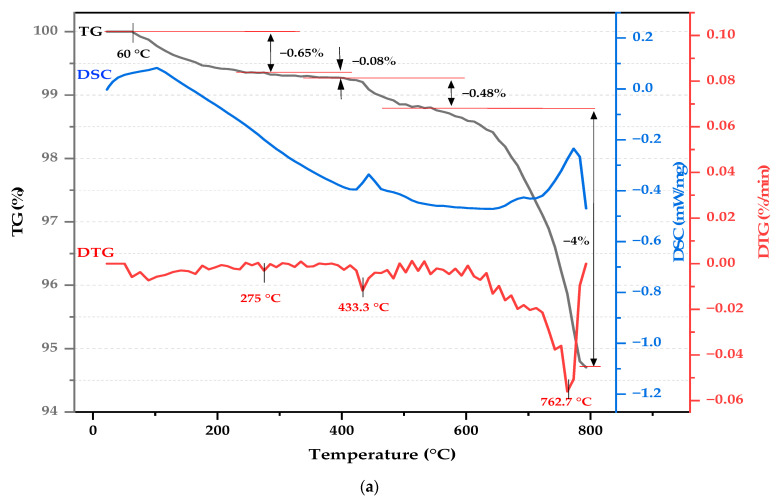

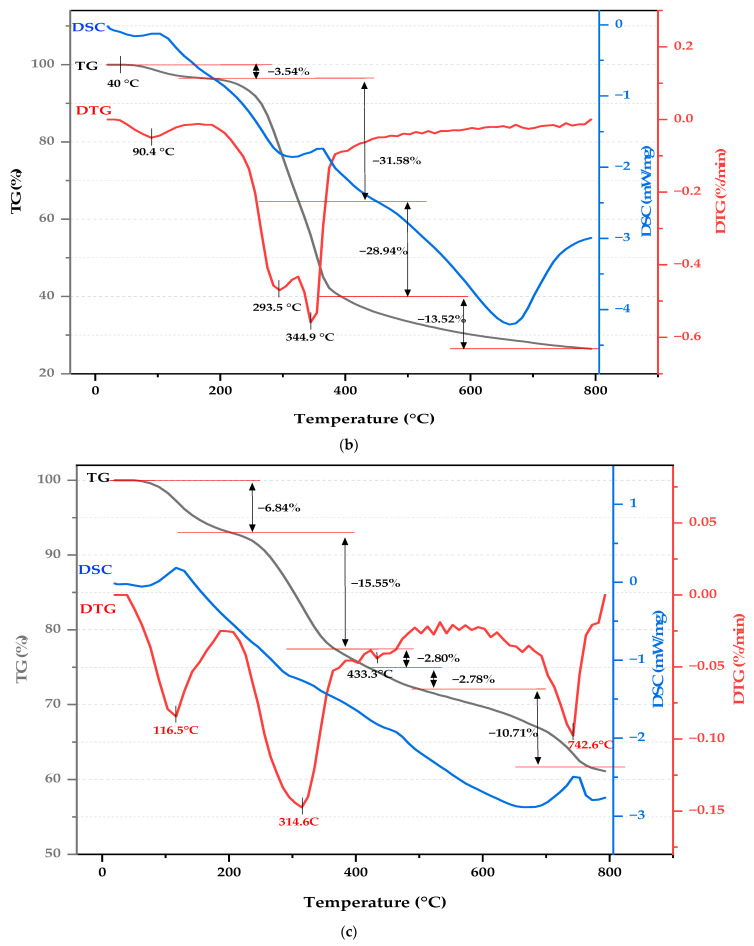
Thermal analysis (TG, DTG, and DSC) of (**a**) Super White Cement, (**b**) Cedar sawdust, and (**c**) WCB (10% SWD).

Figure 7 presents the TG/DTG and DSC analysis results for samples of Super White Cement (Figure 7a), cedar sawdust (Figure 7b), and the WCB 1 board containing 10% cedar sawdust (Figure 7c). The thermal behavior of Super White Cement begins at 60 °C, as shown in Figure 7a, with a total mass loss of 5%. Previous studies [63,70,71] attribute this mass loss to (i) dehydration of crystal-bonded water molecules, primarily due to cement’s hygroscopic nature and the presence of pre-hydrated phases, occurring up to 275 °C; (ii) dihydroxylation of calcium hydroxide (Ca(OH)_2_), as detailed in Equation (1), marked by a moderate-intensity endothermic peak at 433 °C; and (iii) the decarbonation of calcium carbonate (CaCO_3_), as described in Equation (2), indicated by a sharp endothermic peak at 762 °C.

Ca(OH)_2_ → CaO + H_2_O, (1)

CaCO_3_ → CaO + CO_2_, (2)

The TG/DTG and DSC results in Figure 7b indicate that cedar sawdust degradation starts at 40 °C, with a total mass loss exceeding 77%, occurring in three distinct stages. In the first weight loss stage, a 3.54% mass loss is observed, accompanied by a low-intensity endothermic peak at 90.4 °C. This peak is associated with the evaporation of water and light volatile substances from cedar wood. The second stage features two closely connected intense peaks: the first peak, observed at 293 °C, corresponds to a 31% mass loss, primarily due to the decomposition of hemicellulose and minor cellulose dehydration. The second peak, located at 345 °C, shows a 29% mass loss attributed to the thermal decomposition of cellulose [72]. According to Kim et al., wood components decompose at different temperatures: hemicellulose between 150 and 350 °C, cellulose between 275 and 350 °C, and lignin between 250 and 500 °C [73]. The final phase, starting at 390 °C, reveals a 13% mass loss, likely due to carbonaceous residues’ combustion and lignin’s thermal degradation, which is relatively stable and degrades over a wider temperature range [72].

The TG/DTG and DSC curves of the WCB 1 shown in Figure 7c reveal a total mass loss of 38%, occurring in several stages marked by significant endothermic effects. The initial endothermic effect observed between 40 and 190 °C is attributed to the dehydration of interstitial water and the moisture present in cement and sawdust, as well as to the thermal decomposition of calcium silicate hydrate (C–S–H) and ettringite [74,75]. According to the findings of Yel et al. [71], the largest mass loss (15%) observed between 190 and 390 °C, is attributed to the decomposition of wood components, during which the most intense endothermic peak occurs, consistent with findings from cedar sawdust analysis in Figure 7b. Between 420 and 490 °C, a low-intensity thermal effect is associated with the dehydration of calcium hydroxide (Ca(OH)_2_), accompanied by a measured mass loss (approximately 2.78%) that is slightly lower than reported in other studies [71,74]. Petkova et al. suggest that this discrepancy may indicate a lower presence of portlandite crystals, potentially leading to the increased amount of C-S-H phases in the samples and enhanced strength properties [63].

The decarbonization of calcium carbonate (CaCO_3_) occurs between 620 and 800 °C, marked by an intense endothermic peak and a mass loss exceeding 10%. Similar observations have been noted in previous studies on cement-bonded particleboards made from various species [71,74,75]. The higher mass loss (10.7%) compared to raw cement (4%) in this temperature range suggests differences in the amount and crystallization of carbonates formed. Studies on accelerated carbonation in particleboards, including Cabral et al.’s research [74], indicate a higher mass loss in carbonated samples due to the decomposition of well-crystallized calcium carbonate compared to poorly crystallized forms in control samples. Additionally, Hassan et al. highlight the conversion of portlandite to CaCO_3_ in the presence of organic fibers such as plant fibers [75], suggesting that cedar sawdust promotes increased formation of well-crystallized calcium carbonate within the boards. Similar results were observed by Govin for cementitious pastes containing poplar wood [65].

## 3. Materials and Methods

### 3.1. Materials

**Figure 8:**
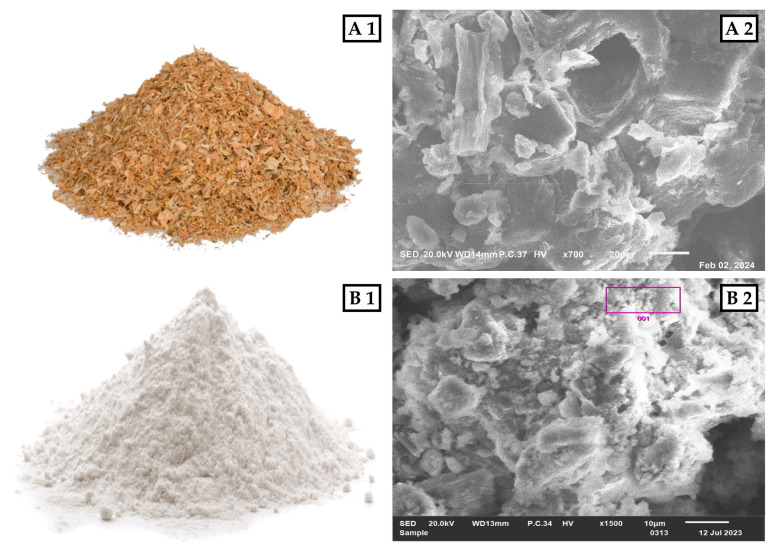
(**A1**) Image of cedar sawdust; (**A2**) SEM image of cedar sawdust; (**B1**) Image of SWC; (**B2**) SEM image of SWC.

The materials used in the production of the boards included cedar sawdust as a reinforcing agent, Super White Cement (SWC) as a binder, and an appropriate amount of water. Figure 8 illustrates the morphologies and images of the cedar sawdust and cement employed in this study.

#### 3.1.1. Cedar Sawdust

The raw material used in this study comprised wood sawdust sourced from woodworking workshops in Khénifra, Morocco, where Atlas cedar wood is commonly processed. Following the cutting of cedar logs, the resulting residues underwent collection under industrial conditions, requiring the removal of flakes and the sorting of the fraction suitable for the study. The sorted sawdust particles then underwent classification using 2 mm and 4 mm diameter sieves. Particles that passed through the 4 mm mesh but were retained by the 2 mm mesh were chosen for the study. Any particles larger than 4 mm were trimmed before reuse, while those smaller than 2 mm were deemed insignificant and excluded. The acquired sawdust particles underwent air-drying for two weeks until reaching a moisture content of 12%. Subsequently, their density was determined, and they were stored in plastic bags for future utilization.

#### 3.1.2. Super White Cement (SWC)

The Super White Cement (SWC, CEM 52.5R) utilized as a binder was a commercial product from the manufacturer Çimsa (Ataşehir, Turkey). The cement was characterized following the procedures outlined in NM 10.1.005 (2008) and EN 196-2-3-6 standards [76,77,78,79]. The characterization process involved analyzing the chemical composition of cement samples using X-ray fluorescence spectroscopy. Each sample, weighing 10 g, was compacted inside a metal ring using an APM device, alongside specific admixture tablets. The resulting pellet was then inserted into an XRF spectrometer (ARL 9900, Thermo Fisher Scientific, Waltham, MA, USA) for analysis. Based on the chemical composition of the SWC, Bogue’s equations were employed to calculate the percentage of anhydrous cement compounds [80]. Additionally, cement characterization encompassed the determination of physical properties, mainly specific surface area, setting time, fineness, density, and loss on ignition. The obtained cement composition and its physical properties are displayed in Table 2.

### 3.2. Boards Production

The production of the boards was based on a single variable, namely sawdust content by weight. Six different levels of cedar sawdust content were employed: 10%, 12.5%, 15%, 17.5%, 20%, and 25%, with four repetitions for each cedar sawdust content level, resulting in a total of 24 panels. The quantities of components and the designations of the prepared panels are indicated in Table 6. Methods from prior studies were employed to calculate the required amounts of cement and cedar sawdust [22,44], aiming to produce boards with a density of 1200 kg/m^3^ and nominal dimensions of 28 cm × 28 cm × 1 cm. The specifics of the components and designations of the prepared boards are provided in Table 6, along with the necessary water amounts calculated using Equation (3), developed by Simatupang [81], as follows:

Water requirement (mL) = 0.35 Z + (0.30 − r) × H,(3)

where Z (g) represents the cement weight, r (%) indicates the moisture content of the sawdust, and H (g) denotes the sawdust weight.

**Figure 9:**
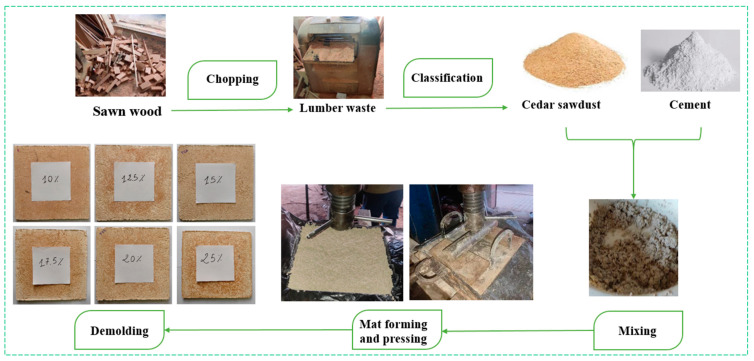
The production steps of WCB.

**Figure 10:**
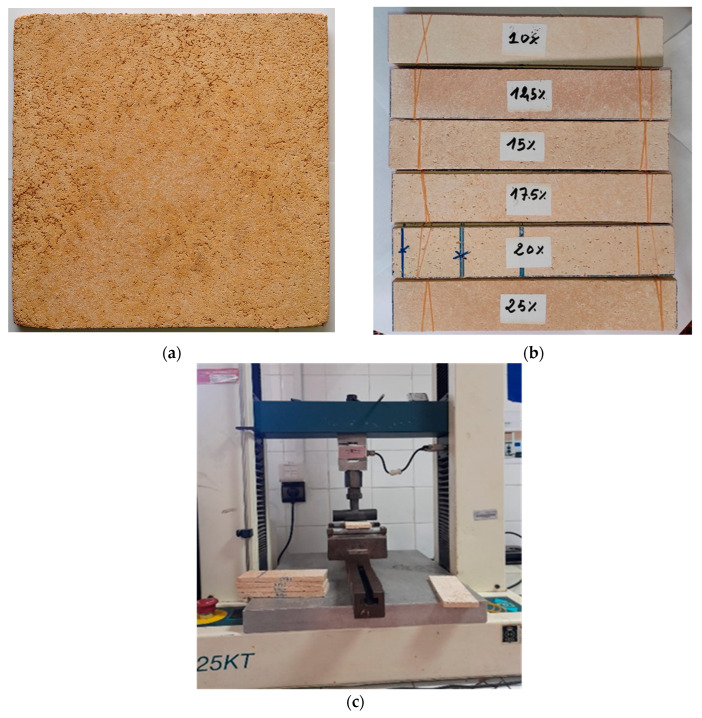
(**a**) WCB5 after finishing operations; (**b**) MOR-MOE test samples; (**c**) The universal testing machine used for mechanical characterization.

The board production process is illustrated in Figure 9. Following weighing, the cement and cedar sawdust quantities were meticulously dry-mixed in a plastic bowl before adding the required water amount. The mixture was uniformly stirred for 10 min until achieving a homogeneous paste, free of lumps. The resulting paste was poured into a detachable iron mold designed for creating boards with dimensions of 28 cm × 28 cm × 1 cm. Using a wooden ruler, the paste was evenly distributed to create a flat, homogeneous mat. To aid in demolding and prevent adherence of the board to the mold’s interior, the mat was covered with a polyethylene sheet before placing the metal cap. The paste then underwent 24 h of cold pressing in a manual hydraulic press, with pressure varying between 2 and 6 MPa, to reach a final board thickness of 10 mm. Once pressed, the boards were detached from the mold and stored in polyethylene bags for 28 days to prevent moisture loss, complete the cement hydration, and allow it to fully harden. Subsequently, the boards underwent finishing operations using sandpaper, including sanding the surfaces and edges (Figure 10a). Then, they were cut using circular saws based on the principles specified in the European Standards to obtain specimens suitable for mechanical and physical tests, with the remaining parts utilized in other tests.

### 3.3. Physical and Mechanical Characterization of the Boards

The physical tests encompassed the measurement of density, water absorption (WA), and thickness swelling (TS). A precision balance and a digital caliper were employed to measure the mass and dimensions of the samples following the PNM EN 325 (2012) standard [82]. For density calculations, eight specimens of each content with dimensions of 50 × 50 mm^2^ were used. Average values were obtained by calculating the ratio between the weight (g) and volume (mm^3^) of each test specimen according to the EN 323 (1993) standard [83]. The WA and TS were evaluated successively after both 2 h and 24 h immersion in water. Tests were manually conducted on square specimens, similar to those used for density measurements, following the procedures described in the EN 317 (1993) standard [84]. Eight samples were tested for each cedar sawdust content level by immersing them in deionized water under room conditions. The initial (W_1_) and final (W_2_) weights before and after immersing were measured, and the WA was calculated using the following formula:

Water absorption = (W_2_ − W_1_)/W_1_ × 100. (4)

Similarly, for the initial (T1) and final (T2) thicknesses, the TS was calculated using the formula:

Thickness swelling = (T_2_ − T_1_)/T_1_ × 100. (5)

The assessment of the mechanical properties involved three-point bending tests conducted on rectangular specimens measuring 25 × 5 × 1 cm^3^ (Figure 10b). For each cedar sawdust content level, six samples were individually mounted on a universal testing machine (H25KT, Tinius Olsen, Horsham, PA, USA) equipped with a 25 KT compression/tension sensor (Figure 10c).

A perpendicular bending load was applied to the center of each sample at a speed of 10 mm/min using an electromechanical motor until failure occurred. The modulus of rupture (MOR) and the modulus of elasticity (MOE) were determined following the EN 310 (1993) standard [85].

### 3.4. Microstructural Analysis of the Boards

Microstructural analysis of the produced boards was conducted via scanning electron microscopy (SEM) using a JSM-IT500HR/LA microscope (JEOL, Tokyo, Japan). This analysis was conducted to investigate structural variations in the boards. It also examined the differences in the fracture surfaces between boards containing 10% and 25% cedar sawdust after bending tests. The SEM analysis was performed at an accelerating voltage of 10 kV and a working distance of 13.4 mm in a low-vacuum mode (LV-SEM). The SEM instrument is coupled with Energy-dispersive X-ray spectroscopy (EDS), which was utilized to examine variations in the elemental composition of the samples.

### 3.5. Fourier Transform Infrared Spectroscopy Analysis

Fourier transform infrared spectroscopy (FTIR) was employed to observe chemical reactions and identify functional groups of cedar sawdust, Super White Cement, and various WCB samples. Spectral data were acquired using a Nicolet iS10 FTIR spectrophotometer (Thermo Fisher Scientific, Waltham, MA, USA), utilizing the Attenuated Total Reflectance (ATR) technique. Pellets were prepared from 300 mg of finely ground sample powders, ensuring an accurate representation of each material. Spectra were recorded over a broad wavelength range spanning from 500 to 4000 cm^−1^, with a high spectral resolution of 4 cm^−1^ and an average of 32 co-addition scans.

### 3.6. Thermal Analysis of the Boards 

Thermogravimetric analysis (TGA), derivative thermogravimetry (DTG), and differential scanning calorimetry (DSC) were performed to investigate the thermal behavior and stability profiles of cedar sawdust, Super White Cement, and related WCBs. The measurements were conducted using a simultaneous thermal analyzer, specifically the Netzsch STA 449 F5 Jupiter (NETZSCH Gerätebau GmbH, Selb, Germany). Operational parameters for TG/DTG-DSC measurements included a sample mass of 15.0 ± 5.0 mg and an Al_2_O_3_ sample crucible with a volume of 85 µL. Heating was carried out under static conditions in an argon atmosphere, with a temperature range spanning from 25 °C to 800 °C and a heating rate of 10 °C/min.

### 3.7. Statistical Analysis

Statistical analysis was carried out on the data obtained during the experiment, including a one-way analysis of variance (ANOVA), to assess the relative importance of various sources of variation in the physical and mechanical property results. The ANOVA revealed statistically significant differences between the levels of all properties. Subsequently, mean values were compared using Tukey’s test at a 95% confidence level.

## 4. Conclusions

This study highlights the feasibility of using cedar sawdust as a reinforcing agent in wood–cement boards, providing an effective approach for recycling bio-waste and advancing eco-friendly construction materials. Incorporating cedar sawdust improves production efficiency and environmental benefits, with optimal physical and mechanical performance achieved at a sawdust content of 10%. The key findings from this research include the following:

- Atlas cedar sawdust, with lower extractives compared to other species, enhances board properties without significantly affecting the cement setting.
- Physical properties indicate that board density decreases with increasing cedar sawdust content, reaching its lowest at 25% sawdust. Concurrently, water absorption and thickness swelling increase with higher sawdust content due to its hygroscopic nature and resultant porosity.
- Mechanical properties reveal that boards with 10% cedar sawdust content exhibit the highest modulus of rupture (MOR) and modulus of elasticity (MOE). In contrast, increasing the sawdust content beyond this level results in lower mechanical performance due to higher porosity and insufficient cement bonding, making the boards less suitable for structural applications.
- Morphological analysis shows that lower sawdust content (10%) provides better adhesion between the sawdust and the cement matrix, resulting in higher density and improved mechanical properties. In contrast, boards with higher sawdust content (25%) display increased porosity and weaker particle–matrix bonds, contributing to reduced strength and increased water absorption and thickness swelling.
- EDS analysis indicates an increased calcium-to-silicon ratio at the particle–matrix interface, suggesting enhanced bonding for boards with 10% cedar sawdust due to the accumulation of cement hydration products, mainly calcium hydroxide (Ca(OH)_2_) and calcium silicate hydrate (C-S-H).
- FTIR spectroscopy results show an increase in calcium carbonate and hydroxide formation, with higher sawdust content, indicating improved carbonation and C-S-H gel formation.
- Thermal analysis confirms the consistent formation of well-crystallized calcium carbonate and a lower presence of portlandite crystals, contributing to increased C-S-H phases in the boards.

This research underscores the potential of using locally sourced bio-waste and highlights the need for further investigations to optimize board performances. Future studies will focus on strategies to decrease porosity at higher sawdust contents and the use of pretreated cedar sawdust, and explore chemical additives to reduce setting time and improve the interface bonding quality. Addressing these aspects will enhance the sustainability and practical application of wood–cement boards, promoting more environmentally friendly construction practices.

## Figures and Tables

**Table 1:** Atlas cedar wood chemical composition found by Quiquandon [26].

Components	Content (%)
Cellulose	50
Hemicelluloses	12.10
Lignin	32
Resin	1.45
Extractives	4.24
Ash	0.31

**Table 2:** Cement composition and its physical properties.

Chemical Composition		Physical Properties	
SiO_2_ (%)	20.43	Specific surface Blaine (cm^2^/g)	4500
Al_2_O_3_ (%)	4.21	Initial setting (min)	100
Fe_2_O_3_ (%)	0.43	Final setting (min)	130
CaO (%)	66.03	Refusal at 45 µm sieve (%)	1.69
MgO (%)	1.40	Refusal at 90 µm sieve (%)	0.18
SO_3_ (%)	3.35	Apparent density (g/L)	3047
K_2_O (%)	0.54	Loss on ignition (%)	3.45
Na_2_O (%)	0.14		
**Bogue’s Compounds**			
C_3_S	84.59	C_2_S	5.22
C_3_A	10.43	C_4_AF	1.30

**Table 5:** Estimated elemental chemical composition for the composites.

	Detection of Chemical Elements (% by Weight)				Ca/Si
	C	O	Si	Ca	
Cedar sawdust	52.38	47.62	-	-	-
Surface of the cement matrix	8.28	51.12	8.97	31.63	3.52
Surface of reinforcement	41.18	53.57	1.04	4.21	4.05

**Table 6:** The production plan of wood cement boards.

Board Types	Raw Material		
	Sawdust (wt%)	Cement (wt%)	Water (g)
WCB 1	10	90	313
WCB 2	12.5	87.5	309
WCB 3	15	85	305
WCB 4	17.5	82.5	301
WCB 5	20	80	297
WCB 6	25	75	289

## Data Availability

Data are contained within the article.

## Abbreviations

WCB	Wood-Cement Board
WA	Water Absorption
TS	Thickness Swelling
SEM	Scanning Electron Microscopy
EDS	Energy-Dispersive X-ray Spectroscopy
XRF	X-ray Fluorescence
TG/DTG—DSC	Thermal analysis
FTIR	Fourier Transformed Infrared
CBPB	Cement-Bonded Particleboard
SWC	Super White Cement
WCB 1	Boards with 10% Cedar sawdust
WCB 2	Boards with 12.5% Cedar sawdust
WCB 3	Boards with 15% Cedar sawdust
WCB 4	Boards with 17.5% Cedar sawdust
WCB 5	Boards with 20% Cedar sawdust
WCB 6	Boards with 25% Cedar sawdust
C–S–H	Calcium silicate hydrate
SWD	Cedar sawdust
